# Correlation between functional disability and quality of life in patients with adhesive capsulitis

**DOI:** 10.1590/1413-78522015230200791

**Published:** 2015

**Authors:** Marcos Rassi Fernandes

**Affiliations:** 1 Universidade Federal de Goiás, Faculdade de Medicina, Department of Orthopedics and Traumatology, Goiânia, GO, Brazil, 1. Department of Orthopedics and Traumatology, Faculdade de Medicina da Universidade Federal de Goiás, Goiânia, GO, Brazil

**Keywords:** Quality of life, Shoulder, Bursitis, Shoulder pain

## Abstract

**OBJECTIVE:**

To determine the correlation between functional disability and quality of life of patients with adhesive capsulitis.

**METHODS:**

Two instruments (WHOQOL-BREF and DASH) were applied to evaluate the quality of life and functional capacity of patients with adhesive capsulitis. Inclusion criteria were age between 35 and 75 years old and achievement of shoulder imaging. Each domain of the WHOQOL-BREF was correlated with DASH. Pearson's correlation coefficient was used for parametric variables and Spearman's correlation coefficient was used when at least one variable had a non-normal distribution. The level of significance was p <0.05.

**RESULTS:**

Forty three patients with mean age of 54.7 years old were evaluated. The mean values found in the physical, psychological, social and environmental domains of the WHOQOL-BREF and DASH were 45.3, 63.9, 68.2, 60.2 and 61.6, respectively. A moderate negative correlation was found between DASH and the physical domain of WHOQOL-BREF (r= - 0.583*, p*<0.001).

**CONCLUSION:**

The only domain where WHOQOL-BREF correlates with DASH is the physical domain, suggesting that measures to promote the improvement of functional capacity may lead to better quality of life of patients with adhesive capsulitis*.*

**Level of Evidence IV, Prospective Study.:**

## INTRODUCTION

Adhesive capsulitis is a disease with a prevalence of 2 to 5% in the general population. It is characterized by disabling pain and active and passive restriction of shoulder movements. The diagnosis is primarily clinical, based on the criteria described by Codman. It affects more women (2: 1) in the age group of 40 to 60 years old, but without preference for side or dominance.^1^

It may appear on the primary form or secondary to other diseases such as diabetes and hypothyroidism.^2^ It occurs distinctly in three phases: hyperalgesia, freezing and defreezing.^3^ However, its resolution can range from two to seven years.^3^^-^ 5 By presenting a chronic course and unwieldy treatment, this condition affects both shoulder function for daily living activities, compromising the quality of life (QoL) of patients, such as washing their back, make the bed and pull the car seat belt with the affected limb.^4^

The impact assessment of orthopedic conditions such as adhesive capsulitis has been restricted to clinical and functional aspects. Such assessments do not allow considering all the implications that the disease can cause to the patient's life. There has been a growing interest among researchers for the use of tools that allow a more holistic assessment, based on the patient's information about their health condition, which would examine not only the natural history of the disease, but also the assessment of its QoL in the physical, emotional and social aspects.^6^

A general tool for assessing QoL still little used in orthopedics is the short version of the Quality of Life World Health Organization test (WHOQOL-BREF), consisting of 26 questions in four domains (physical, psychological, social relationships and environment), which combined reflect a comprehensive score, which ranges from zero to 100, the higher the score, the better the QoL.^7^ Its main limitation is that it does not focus on issues relevant that the disease would harm the patient's QoL such as dressing, writing or opening a dor.^8^ It is, thus, necessary another more specific assessment.^6^

The Disabilities of the Arm, Shoulder, and Hand Questionnaire (DASH) is a regional questionnaire consisting of 30 questions, rather specific to evaluate the functional capacity of the affected upper limb, also being self-administered.^9 ^It has a score that ranges from zero to 100, the higher the score, the greater the functional disability. DASH has been recommended in the evaluation of patients with shoulder disabilities.^6^ Both DASH and WHOQOL-BREF were validated in the Portuguese language.^7^^,^ 9

Although the decreased shoulder function has been well established in adhesive capsulitis studies,^1^^-^ 5 further research is needed to understand the impact of this disease on the variable QoL. The objective of this study was to test our hypothesis that QoL is correlated with shoulder functional disability in patients with adhesive capsulitis.

## METHODS

A prospective cohort study of patients with adhesive capsulitis, describing the correlation between the instruments to assess functional capacity and quality of life was held.

Participant patients were selected during routine consultations at a specialized office, located in an orthopedic hospital, from August 2010 to February 2012.

Adhesive capsulitis was clinically diagnosed as: presence of constant pain, severe (zero points in Constant scale)^10^ and prolonged (more than four weeks), with limited active and passive shoulder movements, with an anterior elevation to 130°, external rotation to 50° and internal rotation up to L5.^1^

Inclusion criteria were: clinical diagnosis of adhesive capsulitis; patient oriented in time and space and having cognitive conditions to participate; aged between 35 and 75 years old; having performed shoulder radiographs in three views (real AP, axillary profile and scapular profile) and magnetic resonance imaging in the last 30 days; not be concurrently undergoing another treatment for adhesive capsulitis; not having done subacromial infiltration in less than 15 days; glycosylated hemoglobin lower or equal to 7% in case of diabetes associated and consent to participate in the study, by signing the Free and Informed Consent Term.

Patients with the following conditions were excluded: concomitant diseases such as full rotator cuff injury, instability, glenohumeral arthrosis and locked dislocation of the shoulder; having stroke sequelae (hemiplegia or paresis); recent breast surgery (1 month); receiving chemotherapy or radiotherapy; with bilateral involvement of the disease and previous surgery on the affected shoulder.

The instruments WHOQOL-BREF^7^ and DASH^9^ were applied to each selected patient. The questionnaires were self-administered without the interference of the interviewer. In order to classify the disease and its severity, we used the Zuckerman classification.^11 ^After the diagnosis was established, patients were treated with serial blocks of the suprascapular nerve.

The protocol of this study was approved by the Research Ethics Committee "Dr. Henrique Santillo" Suleide - SES / GO on June 23, 2010. 

### Data analysis

Data were recorded in an Excel 2010 spreadsheet and analyzed by the Statistical Package for the Social Sciences (SPSS 20.0). Quantitative variables were expressed as mean, median and standard deviation, while the qualitative variables are in absolute numbers and percentages.

The distribution of absolute frequencies observed in the qualitative variables was analyzed using the chi-square test. For quantitative variables, we used the Shapiro-Wilk normality test, considering the sample size less than 60. Correlation analysis was used to describe the strength (degree) and the direction of the relationship between variables; Pearson and Spearman correlation coefficients were used according to the distribution type identified for each variable. The probability of rejecting the null hypothesis was <0.05.

## RESULTS

Forty three patients were analyzed. The mean age was 54.7 years old (range, 40-75) and 53.5% were female. The sociodemographic and clinical data of patients with adhesive capsulitis are shown in Table 1. There were differences in the distribution of the variables race (p <0.001), dominance (p <0.001), severity (p <0.001) and classification (p=0.047).

**Table 1.** Sociodemographic and clinical characteristics of the sample.

Variables		n	%	p*
Age	Mean 54.65			
	Median 54			
	SD ±8.97			
	Minimum 40			
	Maximum 75			
Gender	Feminine	23	53.5	0.647
	Masculine	20	46.5	
Race	White	34	79.1	0.000
	Not white	09	20.9	
Side	Right	18	41.9	0.286
	Left	25	58.1	
Dominance	Right handed	41	95.3	0.000
	Left handed	02	4.7	
Classification	Primary	15	34.9	0.047
	Secondary	28	65.1	
Severity	Mild	10	23.3	
	Moderated	27	62.8	0.000
	Serious	06	14	

* Chi-square test. Source: Medical files.

The mean scores and standard deviation of the four domains of the WHOQOL-BREF generic instrument (physical, psychological, social relationships and environment) and the DASH questionnaire, as well as the results of their normality test are shown in Table 2.

**Table 2.** Mean scores and normality tests of WHOQOL-BREF and DASH domains on subjects with adhesive capsulitis. (n=43)

Domain	Mean	Median	Min - Max	SD	CI	p
Physical	45.34	46.42	11 - 86	19.75	39.27- 51.43	0.331*
Psychological	63.95	66.66	25 - 88	16.33	58.93- 68.92	0.009
Social	68.21	66.66	17 - 100	19.26	62.29- 74.15	0.024
Environmental	60.24	62.50	19 - 91	15.62	55.44- 65.06	0.350*
DASH	61.68	64.16	16 - 100	18.71	55.92- 67.44	0.510*

Min: minimum; Max: maximum; SD: Standard deviation; CI: Confidence interval; *normal distribution. Source: Medical files.

**Figure 1.**
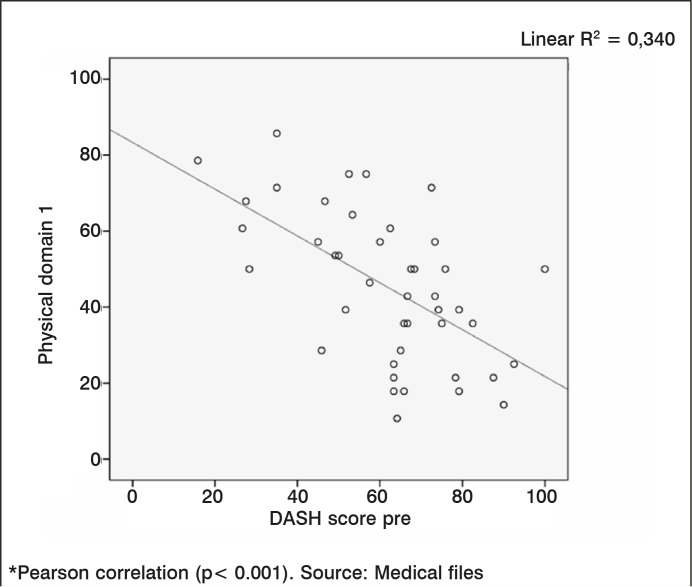
Correlation between DASH and WHOQOL-BREF physical domain in subjects with adhesive capsulitis (n=43).

The Pearson correlation coefficient was used between the DASH variables x physical domain (r = - 0.583 / p <0.001) and DASH x domain environment. However, the Spearman correlation coefficient was used between the DASH variables x psychological domain and x DASH social relationships domain. (Table 3) Therefore, the only domain of the WHOQOL-BREF instrument that correlated with the DASH was physical, being a negative correlation; the greater the patient's functional disability, the lower his/her QoL. (Figure 1)

**Table 3.** Correlation DASH x WHOQOL-BREF.

	Physical*	Psychological**	Social relationships**	Environmental*
DASH	r= - 0.583	r= - 0.260	r= - 0.199	r= - 0.292
	p= 0.000	p= 0.092	p= 0.201	p= 0.057
(95% IC)	- 0.739 a - 0.378	- 0.484 a 0.026	- 0.478 a 0.085	- 0.521 a 0.070

*Pearson correlation; ** Spearman correlation; CI: Confidence interval; Source: Medical files.

## DISCUSSION

Adhesive capsulitis is a disease that affects the shoulder joint on the clinical and functional aspect, however it is unknown whether it can affect the whole quality of life of patients. Self-administered questionnaires are, therefore, needed as required outcomes to assess the effect of impairment in physical, social and psychological life of these individuals.^12 ^Patients' perception of the impact of the disease in their health is gaining more and more emphasis on scientific literature,^12^^-^ 14 which justifies this research due to the interest in the study of the variable quality of life. The wide individual variation in DASH scores from 16 to 100, with an average of 61.6, translates to the health professional that there are cases with little disability and other with high functional disability of the affected shoulder, while the average of 45.3 in the physical domain of the WHOQOL-BREF instrument points out that patients with this disease also have low QoL.

In all domains assessed by WHOQOL-BREF the average quality of life was over 60 and considering only the physical domain this average was reduced, reflecting the impact of the disease on patients' lives. The fact that this instrument is not specific to assessments related to functional capacity may explain the higher averages in those domains in which other aspects such as the psychological and social have been evaluated.

The adoption of these validated self administered questionnaires was the most important innovation in the last decade, and its use is supported in science, especially for monitoring the health status of people with musculoskeletal disorders. Instruments based on patients opinion are usually easy to be filled, being fast and cheap.^7^^,^ 9 ^,^ 15 The study of the impact of adhesive capsulitis in the quality of life of patients with WHOQOL-BREF and DASH questionnaires applied simultaneously represents a contribution to science, since little is known about their correlation specifically in this disease.

It is important to report that WHOQOL-BREF is a practical questionnaire with good psychometric performance that evaluates the status of patient's general health and quality of life, but it does not include specific questions on the shoulder.^7^ However, DASH is a reliable instrument with internal consistency that assesses the symptoms and functional status of patients with upper limb disorders.^9^^,^ 16 ^,^ 17

The following pathologies: rupture of the rotator cuff, rheumatoid arthritis and carpal tunnel syndrome have already been studied from the perspective of general and specific instruments.^15^^,^ 18 ^,^ 19 In line with the literature, we consider important to use two questionnaires to assess QoL and functional capacity of affected individuals, and WHOQOL-BREF provided a broader view of health, while DASH had higher sensitivity to reflect the clinical condition of the disease.^7^^-^ 9 ^,^ 16

The results of this study showed a moderate correlation between DASH and the physical domain of WHOQOL-BREF. This indicates that the shoulder disability in adhesive capsulitis has similar effects on the physical aspect of QoL. However, it is important to emphasize that study on the association between variables does not imply a cause and effect one another. It only indicates and signals the direction and the magnitude of this correlation.

When a researcher wants to know whether an instrument assesses what he really wants to evaluate, he compares it with other similar instruments to verify convergence or correlation between them.^20^ That was the case in this study between DASH and the physical domain of WHOQOL BREF, since the two questionnaires assess whether there is any impairment in physical function of the affected region. Therefore, the individual with adhesive capsulitis, while filling these instruments is telling his doctor that he has an important functional disability to perform his activities of daily life, such as dressing or preparing a meal, as well as to do his work.

The same cannot be said regarding DASH and the psychological, social and environment WHOQOL-BREF, which did not show any correlation. The reason would be that the "inability" component of DASH emphasizes primarily on physical function, focusing on musculoskeletal disorders. However, there is a small number of questions in it that assesses the patient's emotional and social function, but not enough to result in some correlation. According to these findings, we can state that DASH does not correlate with instruments of different concepts, i.e., it was not built to assess mental health, nor specific aspects of the disease.^16^^,^ 17

The absence of research literature on correlation between these instruments in individuals with adhesive capsulitis makes it impossible to compare the results of this study. Most orthopedic conditions in clinical practice entails major impact on QoL of the affected patients.^18^^,^ 19 ^,^ 21 Therefore, it makes sense to evaluate the QoL construct, in addition to objective measures such as muscle strength or articular amplitude.^8^ We can report that SF-36 is a general instrument for assessing QoL mostly used in the orthopedic literature, and WHOQOL in its abbreviated form is not recalled by the researches.^12^^,^ 15 ^,^ 16 ^,^ 21 However, it is important to remember that SF-36 does not include specific questions about shoulder.^19^^,^ 22

The novelty about this study is that we can now understand the impact of adhesive capsulitis in the affected individual's QoL from the patient's perspective. The results emphasize that who refers the highest shoulder disability is associated with a lower QoL, which ultimately requires a special attention by the health professional that assists both his rehabilitation and also social reintegration.

The strengths of the study were the use of instruments for the assessment of both QoL and functional capacity, absence of similar research in science and being prospective. It is also important to consider the fact that adhesive capsulitis have been diagnosed by clinical examination and confirmed by imaging studies. Limitations relate to having carried out the study in the two forms of the disease, primary and secondary, regardless of the etiology of hard shoulder, with a non-probability sampling. Further studies should be conducted with larger numbers of subjects with the disease in its most serious form to check the reproducibility of these findings.

## CONCLUSION

The impact of adhesive capsulitis in QoL is worse in the physical domain. The only WHOQOL-BREF domain that correlates with DASH is the physical domain, which is a negative correlation, suggesting that actions aimed at promoting better functional capacity can optimize the QoL of adhesive capsulitis patients.
